# In-Built Fabrication of MOF Assimilated Porous Hollow Carbon from Pre-Hydrolysate for Supercapacitor

**DOI:** 10.3390/polym14163377

**Published:** 2022-08-18

**Authors:** Xin Zhao, Changwei Li, Lei Sha, Kang Yang, Min Gao, Honglei Chen, Jianchun Jiang

**Affiliations:** 1 State Key Laboratory of Biobased Material and Green Papermaking, Qilu University of Technology, Shandong Academy of Sciences, Jinan 250353, China; 2 Key Lab of Biomass Energy and Material, Jiangsu Co-Innovation Center of Efficient Processing and Utilization of Forest Resources, Institute of Chemical Industry of Forest Products, Chinese Academy of Forestry, Nanjing 210042, China

**Keywords:** Co-MOF, pre-hydrolysate, carbon, hydrothermal, supercapacitor

## Abstract

With the fast consumption of traditional fossil fuels and the urgent requirement for a low-carbon economy and sustainable development, supercapacitors are gaining more and more attention as a clean energy storage and conversion device. The research on electrode materials for supercapacitors has become a hot topic nowadays. An electrode material for a supercapacitor, comprising the ZIF-67 in-built carbon-based material, was prepared from a biomass pre-hydrolysate via a hydrothermal process. As a by-product of dissolving slurry, the pre-hydrolysate is rich in carbon, which is an excellent biomass resource. The utilization of pre-hydrolysate to prepare carbon energy materials could realize the high value utilization of pre-hydrolysate and the efficient energy conversion of biomass. Meanwhile, the cobalt-based MOF (such as ZIF-67), as a porous crystalline material, has the advantages of having a regular order, high specific surface area and controllable pore size, as well as good thermal and chemical stability. The addition of ZIF-67 modified the morphology and pore structure of the carbon, and the obtained samples showed outstanding electrochemical performance. One- and two-step synthetic processes generated specimens with a coral-like cross-linked structure and a new type of rough, hollow, dandelion-like structure, respectively, and the pore size was in the range of 2.0–5.0 nm, which is conducive to ion transport and charge transfer. In C2-ZIF-67, the hollow structures could effectively prevent the accumulation of the electrochemical active center, which could provide enough space for the shrinkage and expansion of particles to protect them from the interference of electrolytes and the formation of solid electrolyte interphase film layers. Additionally, the plush tentacle structure with low density and a large specific surface area could expose more active sites and a large electrolyte electrode contact area, and short electron and charge transport paths. Importantly, active, free electrons of small amounts of Co-MOF (1 wt%) could be stored and released through the redox reaction, further improving the electrical conductivity of Carbon-ZIF-67 materials in this work. Consequently, C2-ZIF-67 exhibited superior specific capacitance (400 F g^−1^, at 0.5 A g^−1^) and stability (90%, after 10,000 cycles).

## 1. Introduction

The fast consumption of traditional fossil fuels, such as coal, petroleum, natural gas and so on, is a global issue that may continue to worsen in years to come. The new energy sources, such as wind and solar energy, are becoming increasingly important in order to achieve a low-carbon economy and sustainable development [1]. However, new energy still faces the great challenge of energy storage and conversion, so advanced devices that can store energy are the focus of intensive research [2,3]. In reaction to the ever-increasing request for clean energy innovations, supercapacitors are considered one of the potential photoelectric energy capacity and transformation gadgets for next-generation gadgets and electric vehicles [4]. Supercapacitors (SCs), also known as electrochemical capacitors, have points of interest including a high power density, good cycling performance, high capacitance and long service life [5]. There are two main types of supercapacitors (SCs): electric double-layer capacitors (EDLCs) and pseudocapacitors. The capacitance performance of an EDLC relies on the surface area of the electrode materials due to its capacitance coming from the contact area between the electrode and electrolyte [6]. Carbon has always been utilized as the preferred material for supercapacitors because of its large specific surface area, good chemical stability and strong oxidation resistance [7,8,9]. Biomass carbon material has been widely used because of its low consumption, low pollution and availability of carbons with urea via the in situ doping method. A great deal of previous work has shown that larch-derived carbon with ordered mesoporous structures has been successfully prepared via the soft template method from phenol liquefaction and formaldehyde without a hybridization reaction [10]. However, the above synthesis routes still suffer from some obvious drawbacks such as the amount of formaldehyde and abundant reserves [11]. The biomass-based carbon materials for a supercapacitor electrode were prepared from pre-hydrolysate. The reason why pre-hydrolysate is an excellent raw material for the preparation of carbon materials is due to the abundant carbon content. The use of pre-hydrolysate to prepare carbon materials could realize the high value utilization of pre-hydrolysate and the full utilization of resources. Meanwhile, it provides a new route for the utilization of pre-hydrolysate.

However, the reason for the lower capacitance or energy density of biomass-derived carbon supercapacitors is that their charge storage and ion transfer only occur at or near the surface of electrode materials [12]. It is a concern that focuses on the improving of energy density. As we all know, the energy density of a supercapacitor is basically related to the specific capacitance due to the porous nano-sized capacitance and operating voltage window derived from the rich redox reaction for charge storage.

In addition to looking for a material with high performance as the electrodes, the rational design of the electrode materials’ structure is also an efficient alternative to improve the electrochemical performance [13]. As an advanced material with a metal center and organic block, metal–organic frameworks (MOFs) are considered to be promising for the electrode materials of a supercapacitor, which is attributed to high porosity, large pore volumes and tunable porosities, all of which endow MOFs with tunable functionalities for excellent electrochemical performance [14,15,16]. In particular, taking advantage of its large pore volumes and tunable porosities, MOFs have consequently been utilized as a template to prepare a special morphology and nanostructure with a high surface area owing to the fact that they could regulate ordered structures and offer more actives sites from redox reactions for high capacitance supercapacitors [17,18].

Zeolite imidazole frameworks (ZIFs) have been used as electrode materials for supercapacitors since they have a high specific surface area, high porosity, structure diversity and high chemical stability [19]. ZIF-67, a member of ZIFs, exhibits a regular dodecahedral coordination structure. This structure increases the contact area with the electrolyte for the composite materials [20]. Moreover, ZIF-67 involves the rich N-containing methylimidazole ligand [21], which could provide rich active sites from N and the redox ability from MOF performance, and further enhance the energy density of the supercapacitor. 

Meanwhile, finding a cost-effective and facile method to fabricate composites from biomass-based carbon and MOF is crucial for improving the synergistic effect of electric double-layer capacitors and pseudocapacitive activity. A hydrothermal reaction is a kind of hybrid method which is mild, simple, green and safe [22]. It can not only satisfy the liquid phase conditions of the material but also carry out hybridization efficiently, with regular morphology.

Herein, the obtained ZIF-67/carbon composite materials were prepared by a simple hydrothermal carbonization process and possessed a special hollow structure and hierarchical pore size (2.0–5.0 nm) via the modification and control of ZIF-67. Importantly, the electrochemical performance (400 F g^−1^ at 0.5 A g^−1^) and stability (90% after 10,000 cycles) of the obtained samples are superior to the materials in other works. This work provides a favorable theoretical basis and technical support for the development and utilization of bio-based carbon materials in the field of supercapacitors.

## 2. Experimental Method

### 2.1. Materials Synthesis

#### 2.1.1. Preparation of the Pre-Hydrolysate

Poplar wood (Henglian, Weifang, China) and deionized water (mass ratio = 1:7) were mixed in a pulp digester and then heated for 2 h at 175 °C. The reacted liquid was collected. The homogeneous pre-hydrolysate was obtained by further filtering and centrifuging at 8000 r min^−1^.

#### 2.1.2. Preparation of the ZIF-67 (Co-MOF)

Cobalt nitrate hexahydrate (2.328 g, 99.0%, Sinopharm Chemical Reagent Co. LTD, Shanghai, China) and dimethylimidazole (2.627 g, 98.0%, Sinopharm Chemical Reagent Co. LTD, Shanghai, China) were dissolved in 100 mL anhydrous methanol (99.5%, Sinopharm Chemical Reagent Co. LTD, Shanghai, China), respectively. Subsequently, the two solutions were mixed and stirred for 30 s, and the reaction continued at room temperature without stirring for 24 h. Following this reaction, the purple solid was collected by centrifugal precipitation, then repeatedly washed with anhydrous methanol and vacuum-dried overnight at 80 °C, the obtained purple powder denoted as the Co-MOF (ZIF-67).

#### 2.1.3. Preparation of the ZIF-67/Carbon

Cobalt nitrate hexahydrate (0.470 g), dimethylimidazole (0.530 g) and pre-hydrolysate (100 mL) were mixed and stirred for 30 s at room temperature, and the mixture was heated for 12 h at 250 °C in a Teflon reactor. The mixture was cooled down to room temperature after the reaction, and then, the solid was collected by centrifugal precipitation, repeatedly washed with deionized water and dried at 80 °C. The obtained black solid was carbonized at 800 °C for 2 h with the heat rate of 5 °C min^−1^ under N_2_. The sample was denoted as C1-ZIF-67. This is the one-step synthetic process to prepare the ZIF-67/carbon.

Simultaneously, the prepared ZIF-67 (1 g) and pre-hydrolysate (100 mL) were mixed and stirred for 30 s, then heated for 12 h at 250 °C in a Teflon reactor. Following, the mixture was cooled down to room temperature, and the solid was separated by centrifugal precipitation, repeatedly washed with deionized water and dried at 80 °C. The obtained black solid was carbonized at 800 °C for 2 h with the heat rate of 5 °C min^−1^ under N_2_. The sample was denoted as C2-ZIF-67. This is the two-step synthetic process to prepare the ZIF-67/carbon.

Finally, a certain amount of pre-hydrolysate (100 mL) was stirred for 30 s and then heated for 12 h at 250 °C in a Teflon reactor. The obtained black solid was further carbonized at 800 °C for 2 h with a heat rate of 5 °C min^−1^ under N_2_. The sample was denoted as carbon-hydrolysate and prepared for use as a comparison.

**Figure 1:**
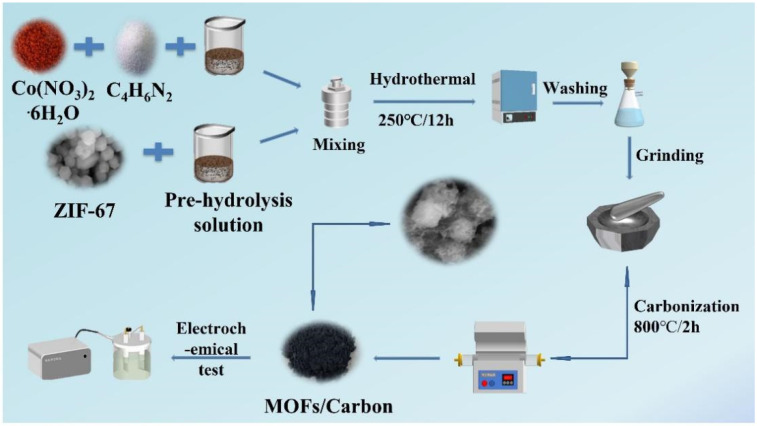
The schematic image of samples.

The schematic image of the whole experimental procedure is shown in Figure 1.

### 2.2. Characterization

The morphologies and pore textures of the obtained samples were investigated using scanning electron microscopy (SEM; JSM-7401F microscope, Hitachi, Tokyo, Japan) operating at an acceleration voltage of 20 kV. Transmission electron microscopy (TEM) images were obtained on a JEOL 2011 apparatus (JEOL, Hokkaido, Japan) operating at 200 kV. The surface composition and chemical state of the obtained samples were investigated by X-ray photoelectron spectroscopy (XPS; Escalab 250Xi, Thermo Fisher Scientific, Waltham, MA, USA) with an Al Kα X-ray source. Powder X-ray diffraction (XRD) patterns of the samples were measured using a Bruker D4 powder X-ray diffractometer (Bruker, Rheinstetten, Germany) with Cu Kα radiation at 40 kV and 40 mA. Raman spectroscopy (Renishaw inVia, London, UK) with a He-Ne laser source (λ = 532 nm) was used to analyze the texture of the MOF/carbon composites. Nitrogen sorption isotherms were measured with a Micromeritics ASAP 2460 sorptometer (Maike, Norcross, GA, USA) using nitrogen as the adsorbate at 77 K. All samples were degassed at 200 °C for more than 6 h prior to the analysis. The surface area (S_BET_) was calculated using the Brunauer–Emmett–Teller (BET) method based on the adsorption data in the relative pressure range of 0.02–0.35, and the total pore volume was determined at the highest relative pressure.

### 2.3. Electrochemical Test

The three-electrode system was used to measure the performance of the ZIF-67/carbon materials in a 1 M KOH aqueous electrolyte. The reference electrode is mercury/mercury oxide (Hg/HgO) and the counter electrode is a platinum sheet. The working electrode was prepared by MOF/carbon, carbon black and polytetrafluoroethylene (mass ratio = 8:1:1). The mixtures (6 mg) were coated onto a 1.5 cm  ×  1.5 cm nickel foam current collector and dried at 60 °C for 8 h. The cyclic voltammetry (CV), galvanostatic charge/discharge (GCD) and electrochemical impedance spectroscopy (EIS) were measured on a PARSTAT 4000 A electrochemical workstation. The specific capacitance of the electrode can be calculated from the galvanostatic charge and discharge curves according to the following equation [23]

*C* = *I*Δ*t*/*m* (1)

Here, *C* is the specific capacitance (F/g), *I* is the current (A), Δ*t* is the discharge time (s) and *m* is the mass of the active material in the electrode (g).

The energy density, *E* (W h/kg), and power density, *P* (W/kg), of each sample were calculated from the discharge plots using Equations (2) and (3), respectively,

*E* = (*C* × Δ*V*^2^)/2 (2)

*P* = *E*/Δ*t* (3)

## 3. Results and Discussion

**Figure 2:**
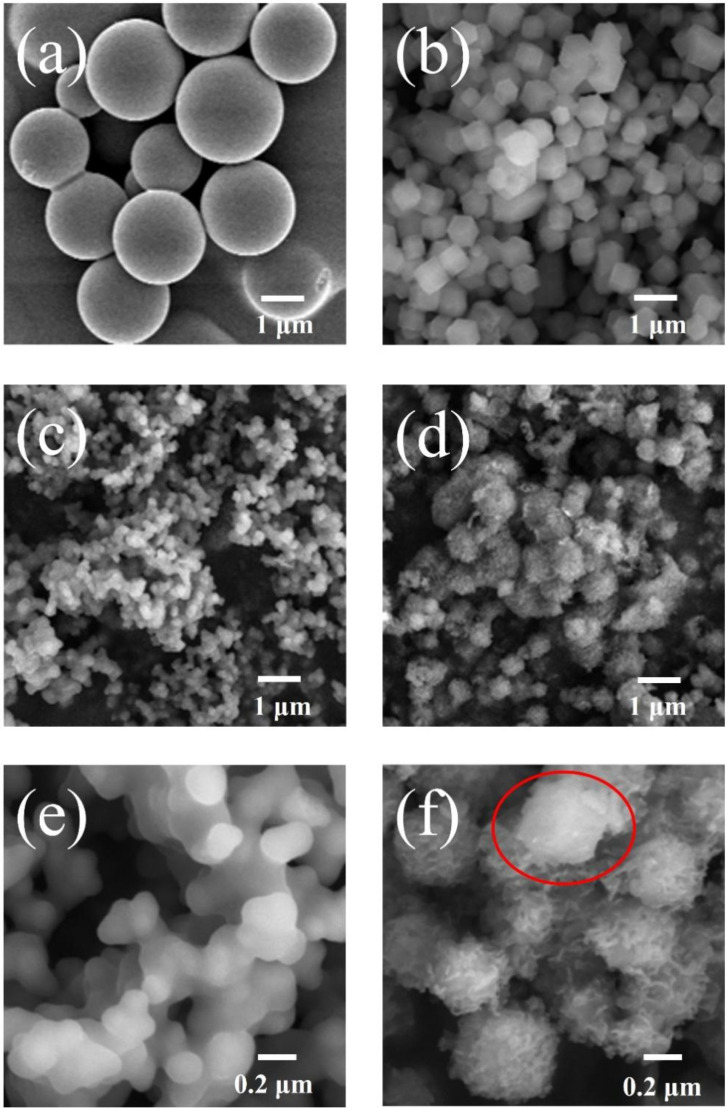
SEM images of the (**a**) carbon-hydrolysate, (**b**) Co-MOF (ZIF-67), (**c**,**e**) C1-ZIF-67 and (**d**,**f**) C2-ZIF-67.

The SEM images of the samples for carbon-hydrolysate, Co-MOF, C1-ZIF-67 and C2-ZIF-67 are shown in Figure 2a–f. In Figure 2a, the sample of carbon-hydrolysate displays a consistent smooth spheres structure with a size of 1–2 μm. The structure is due to the surface energy required for spherical structures being lower and more easily obtained under the hydrothermal conditions. The reason for the different particle sizes is the uneven distribution of different types of sugars in the pre-hydrolysate, including a variety of sugars such as arabinose, ribose and xylose [24]. The Co-MOF (ZIF-67; Figure 2b), which has particle sizes between 0.3 and 0.8 μm, shows a regular dodecahedral coordination structure. What is interesting is that the C1-ZIF-67 and C2-ZIF-67 showed different morphologies from the carbon-hydrolysate and Co-MOF after hydrothermal reaction. The C1-ZIF-67 showed a coral-like cross-linked structure with 0.1 μm particle sizes (Figure 2c), and magnifying the sample image in Figure 2e, the C1-ZIF-67 possessed cross-linking particles with a smooth surface.

However, the C2-ZIF-67 exhibited a rough three-dimensional dandelion-like structures with 0.35–0.90 μm size (Figure 2d), and magnifying the sample image in Figure 2f, the C2-ZIF-67 possessed a plush tentacle on the surface of the dandelion-like structures. The results show that the change in structure and increase in surface roughness of the carbon-based material is due to the introduction of Co-MOF, but the different morphologies of carbon-ZIF-67 is ascribed to the preparation technology, and the one-step and two-step method. As is known to all, double-layer capacitors mainly exist on the surface or near surface where the carbon material is in contact with the electrolyte. Such changes could improve the specific surface area and double-layer capacitance, which is conducive to the improvement in performance for double-layer capacitors, by increasing the surface pore structure and contact surface area. In addition, morphology changes would provide more active sites for oxygen reduction reactions and shorten the transmission path of electrons, which would enhance pseudocapacitance [25].

The formation of C2-ZIF-67, it should be noted, could be carbon growing on the surface of Co-MOF, which could be vaguely found from the red part in Figure 2f.

**Figure 3:**
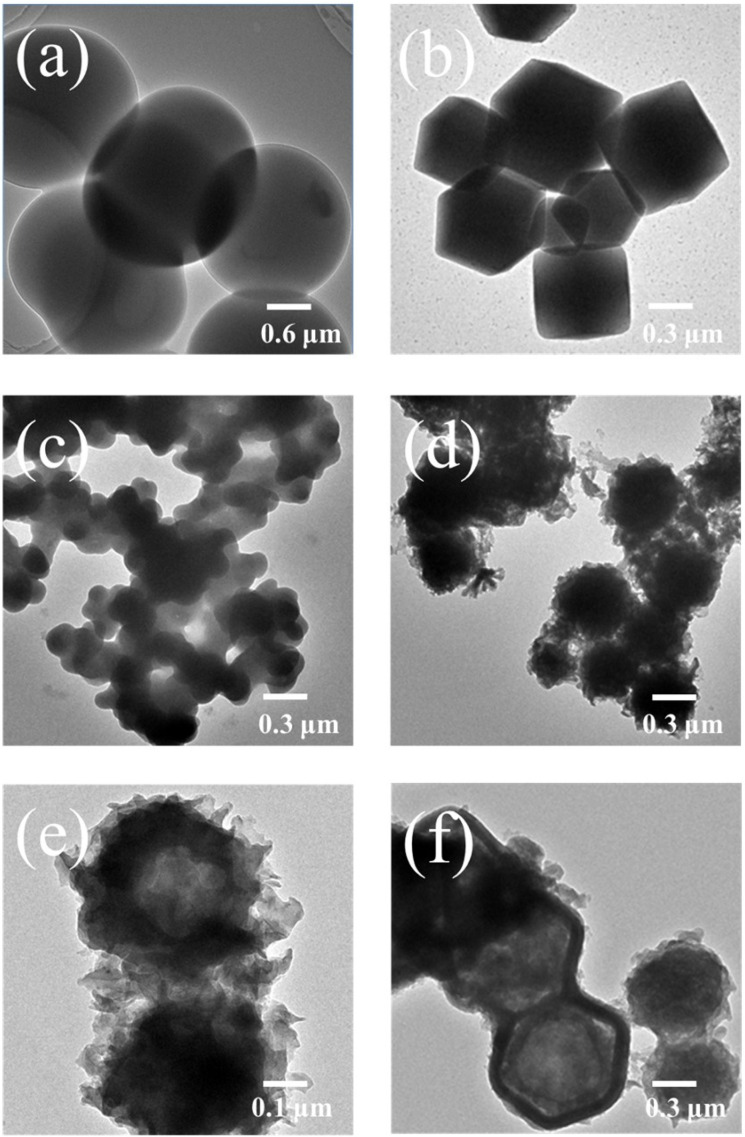
TEM images of the (**a**) carbon-hydrolysate, (**b**) Co-MOF (ZIF-67), (**c**) C1-ZIF-67 and (**d**–**f**) C2-ZIF-67.

The TEM images of the various samples are displayed in Figure 3a–f. The carbon-hydrolysate displayed complete spheres with a 1–2 μm size in Figure 3a, and the Co-MOF (ZIF-67) showed a diamond cube with a 0.3–0.8 μm size in Figure 3b. The C1-ZIF-67 exhibited a cross-linking particle structure with a 0.1 μm particle size, and inside was a solid structure (Figure 3c), but C2-ZIF-67 (Figure 3d–f) showed a hairy and spherical morphology with 0.35–0.90 μm. The formation of C2-ZIF-67 could be from carbon growing on the surface of Co-MOF, which is consistent with the SEM results.

In addition, the inside of C2-ZIF-67 exhibited a hollow structure (Figure 3e,f), which is probably because the inside of the Co-MOF was decomposed to form a hollow structure, and the carbon material was formed on the remaining shell and gradually grew larger to form a dandelion-like structure in the process of carbonization, which was similar to the in-building of Co-MOF into a carbon material. Compared with a solid structure, a hollow structure has the advantages of having a lower density and larger specific surface area, which could expose more active sites and large electrolyte electrode contact area, and short electron and charge transport paths [26]. The cavity could provide sufficient space for the contraction and expansion of particles [27].

**Figure 4:**
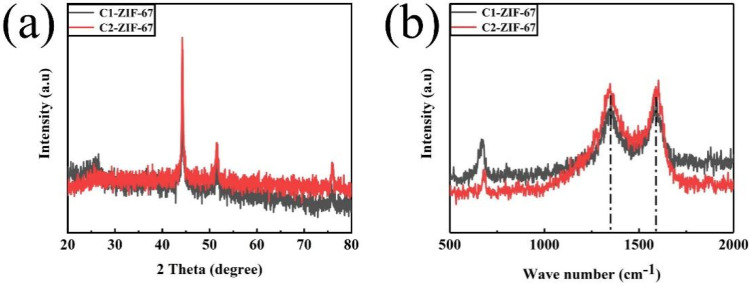
(**a**) XRD patterns and (**b**) Raman of the C1-ZIF-67 and C2-ZIF-67.

Figure 4a shows the XRD patterns of the obtained samples for C1-ZIF-67 and C2-ZIF-67 to confirm their crystal structure. The (002) crystal plane of graphite carbon appears approximately at the 25° peak band for the samples. This result showed that the porous structures and defects of the samples resulted in the low crystallinity and graphitization degree of the carbon materials [28]. The (111), (200) and (220) crystal planes of metal Co (JCPDS No.15-0806) appear in peak bands of approximately 44.3°, 51.7° and 75.9°, respectively. The samples exhibited the same characteristic peaks, which indicated that the samples had the same crystal structure [29]. More interestingly, when the sample was prepared by the two-step hydrothermal method, its characteristic peaks, corresponding to cobalt, exhibited more intensity, which indicated that metallic Co is more active in C2-ZIF-67 than C1-ZIF-67.

The Raman spectrums (Figure 4b) of the C1-ZIF-67 and C2-ZIF-67 displayed two peaks: one was the peak of 1350 cm^−1^ (D band), which reflects the characteristics of disordered graphite, and the other was the G band at 1590 cm^−1^ of graphite carbon. The I_D_/I_G_ value was widely used to reflect the defect degree of graphite materials [30]. The value of both was 1 by calculation, showing that they had the same crystal structure, which was in good agreement with the results of XRD.

**Figure 5:**
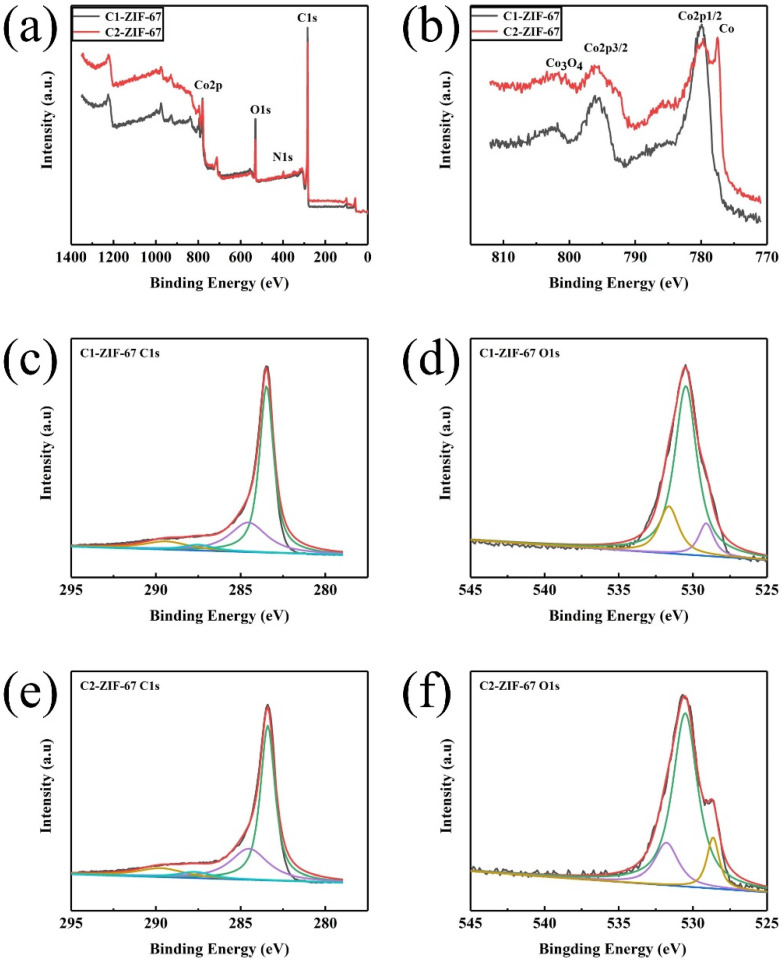
(**a**) XPS spectra of the C1-ZIF-67 and C2-ZIF-67, (**b**) the high-resolution Co 2p XPS spectra of the C1-ZIF-67 and C2-ZIF-67, and the high-resolution XPS spectra of C1-ZIF-67 (C 1s) (**c**), C1-ZIF-67 (O 1s) (**d**), C2-ZIF-67 (C 1s) (**e**) and C2-ZIF-67 (O 1s) (**f**).

In order to determine the chemical composition and type of chemical bonding of the obtained samples, the XPS was tested and the data of the samples for C1-ZIF-67 and C2-ZIF-67 are exhibited in Figure 5. C, N, O and Co are contained in the two samples (Figure 5a). Figure 5b displays the Co spectra of C1-ZIF-67 and C2-ZIF-67. In C1-ZIF-67, the Co 2p_3/2_, Co 2p_1/2_ and Co_3_O_4_ peaks were observed at 795.8, 780 and 803.5 eV, respectively. In addition to the above three peaks, there was another peak at 777.5 eV (metallic Co) in C2-ZIF-67. In the C 1s spectrum of the C1-ZIF-67 sample (Figure 5c), there were four peaks at 283.4, 284.5, 287.5 and 289.4 eV, which represent C-C sp^2^, C-C sp^3^, C-O and C-N bonds, respectively. The C 1s spectrum of the C2-ZIF-67 sample exhibited similar bonds to C1-ZIF-67, with C-C sp^2^ (283.4 eV), C-C sp^3^ (284.5 eV), C-O (287.7 eV) and C-N (289.7 eV) bonds, respectively (Figure 5e). The three Gaussian peaks at 531.6, 530.5 and 529.1 eV are shown in the O 1s spectrum of the C1-ZIF-67 sample (Figure 5d) due to the presence of the C-O bonds, adsorbed -OH groups on the surface and lattice oxygen in Co_3_O_4._ Peaks similar to the sample of C1-ZIF-67 were presented at 531.7, 530.4 and 528.7 eV of the C2-ZIF-67 sample (Figure 5f). Actually, oxygen groups with high electronegativity, in addition to providing active sites, could form stable structures by modifying the carbon skeleton [31]. Chemical bonds such as C-C and C-O could enhance the tectonic force and structural stability. Moreover, the surface of the carbon material is enriched with oxygen-containing functional groups due to the electronic structures of C and O in the sample being affected by the presence of Co_3_O_4_. In addition, the increase in the capacitance value during electrochemical electronic storage depends largely on this effect.

**Figure 6:**
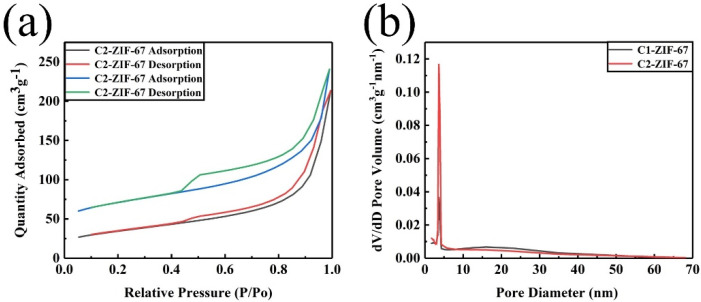
Nitrogen adsorption isotherms (**a**) and the distribution of the pore sizes (**b**) of C1-ZIF-67 and C2-ZIF-67.

Figure 6 shows the nitrogen adsorption and desorption isotherms and pore size distribution curves of the obtained samples for C1-ZIF-67 and C2-ZIF-67. Compared with the C1-ZIF-67, the nitrogen sorption of C2-ZIF-67 increased obviously at various relative pressures, and the hysteresis loop of C2-ZIF-67 obviously changed in a P/P_0_ range of 0.45–1.0 (Figure 6a), which displayed the characteristic mesoporous structure and the higher mesopores’ proportion [32]. The pore size distribution confirmed the multistage porous structure of the materials (Figure 6b). The content of the mesopore region (3.7 nm) was lower in the sample of C1-ZIF-67, while the mesopore content of the C2-ZIF-67 was relatively higher. The pores’ size plays an important role in the supercapacitors, which is attributed to the pore size at the range of 2.0–5.0 nm providing proper channels for ion transport and a greater number of active sites for electrochemical interactions/reactions, which were beneficial to the performance of the supercapacitors [33]. In addition, the micropores contributed to a large space for the adsorption and storage of electrolyte ions, and the macropores were beneficial to the ions’ rapid transfer [34]. This multistage porous structure was of great benefit for the supercapacitors. The textual parameters of the samples are presented in Table 1. The C1-ZIF-67 possessed the lower S_BET_ (121 m^2^ g^−1^), total pore volume (0.209 cm^3^ g^−1^) and S_micro_/S_BET_ ratio (11%). The specific surface area, total pore volume and S_micro_/S_BET_ ratio of C2-ZIF-67 increased to 235 m^2^ g^−1^, 0.254 cm^3^ g^−1^ and 38%, respectively. However, the average pore size (8.4 nm) of C2-ZIF-67 was smaller than the average pore size (11.9 nm) of C1-ZIF-67. These results further indicate that the S_micro_/S_BET_ ratio of C2-ZIF-67 is more favorable to the transmission and storage of supercapacitors, and C2-ZIF-67 is more suitable for supercapacitors than C1-ZIF-67 [35].

**Figure 7:**
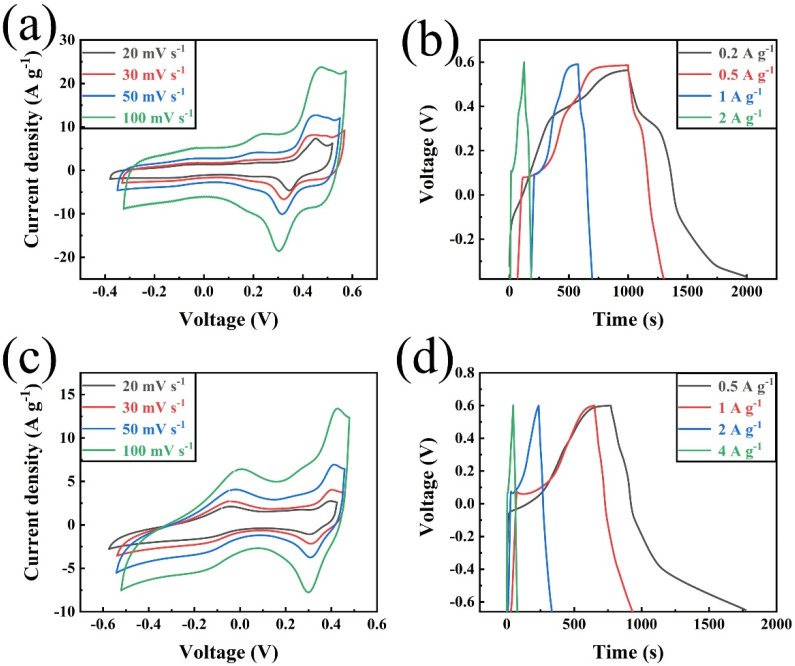
CV curves of C1-ZIF-67 (**a**) and C2-ZIF-67 (**c**) at different scan rates varying from 20 to 100 mV s^−1^, and charge–discharge curves of C1-ZIF-67 (**b**) and C2-ZIF-67 (**d**) at different current densities.

Figure 7 displays the CV and GCD tests of the samples for C1-ZIF-67 and C2-ZIF-67. The CV curves of C1-ZIF-67 and C2-ZIF-67 shown in Figure 7a and c possessed a nearly rectangular shape in the different scan rates, and every CV curve showed a pair of obvious redox peaks, which indicated the synergy of the double-layer and pseudocapacitance behaviors [36]. With the scan rate increasing, the curve shape of C1-ZIF-67 had minor changes, while the curve shape of C2-ZIF-67 had no apparent change, which benefited from low internal resistance (IR) and a hollow structure, and proved that there is little energy wasted during the cycle due to internal resistance. In addition, compared with C1-ZIF-67, the redox peak of C2-ZIF-67 was more obvious and symmetric, which further illustrated the more excellent pseudocapacitive behavior of C2-ZIF-67 [37]. The specific capacitance of C2-ZIF-67 calculated from the discharge plots was 400 F g^−1^ at 0.5 A g^−1^ (Figure 7d), and the specific capacitance of C1-ZIF-67 calculated from the discharge plots was 214 F g^−1^ at 0.2 A g^−1^ (Figure 7b), which verifies our above analysis. The energy density *E* (W h kg^−1^) and power density *P* (W kg^−1^) of the samples were calculated using Equations (2) and (3), respectively. The sample of C2-ZIF-67 produces a high energy density of 80 W h kg^−1^ at a power density of 300 W kg^−1^, and the energy density of C1-ZIF-67 is 42.8 W h kg^−1^, and the power density is 160.5 W kg^−1^. Compared with C1-ZIF-67, C2-ZIF-67 showed a superior electrochemical performance. In fact, a large number of MOF/carbon materials are discussed in Table 2, and the sample obtained (C2-ZIF-67) in this work has a certain advantage in terms of capacitance value. As shown in Table 2, some carbon materials are combined with a single metal in the same process as in this work, such as Ni/C, Zn/C and Co_3_O_4_/C. There are also some samples where a variety of metals are combined with carbon materials, such as Fe-Mg/C and Co-Al/C in the table, but the electrochemical performances of these samples are inferior to the obtained sample in this work. In addition, the preparation process is relatively simple in this work. Importantly, this work realizes the high value utilization of pre-hydrolysate and the full utilization of biomass resources.

**Figure 8:**
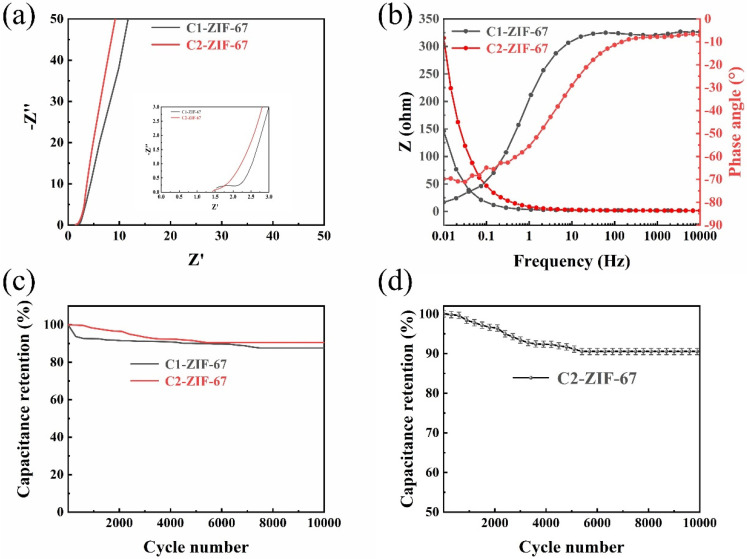
(**a**) AC impedance curve, (**b**) Bode phase angle and electrochemical impedance plots, (**c**) capacitance retention rate after 10,000 cycles for C1-ZIF-67 and C2-ZIF-67 and (**d**) capacitance retention rate after 10,000 cycles for C2-ZIF-67.

Figure 8a displays the representative Nyquist plots of the obtained samples for C1-ZIF-67 and C2-ZIF-67, in which all samples showed a circular arc-like shape in the high-frequency region, and the C2-ZIF-67 sample has the smallest arc radius, which indicates that it has the smallest charge transfer resistance [47]. Due to the diffusion control of the reactants or products for the electrode reaction, a linear shape is presented in the low frequency region [48]. However, the impedance curve deviates from 45° in the low-frequency region, which is possibly due to the induced impedance caused by the uneven surface of the electrode [49]. The slope of C2-ZIF-67 was the largest, indicating that the material exhibited the best conductivity. The C2-ZIF-67 sample exhibits excellent low impedance behavior in the full frequency region. This stems from its special hollow structure, which promotes charge transfer and mass transfer.

Figure 8b presents the Bode phase angle plots of the samples for C1-ZIF-67 and C2-ZIF-67. The C1-ZIF-67 and C2-ZIF-67 have phase angles of approximately −80° and −70° in the high frequency region, respectively. Their phase angles are between −45° (pseudocapacitor) and −90° (ideal capacitor) indicating the presence of intercalation capacitance in the samples [50]. Meanwhile, the capacitance retention of C1-ZIF-67 achieved 87%, and the C2-ZIF-67 reached 90%, after 10,000 cycles (Figure 8c). The result indicates that C2-ZIF-67 has excellent cyclic stability and electrochemical reversibility. Herein, the relatively high retention rate (90%) of C2-ZIF-67 indicates that it has advantages as an energy storage material, possibly because its ratio of microporous and mesoporous structures provides efficient ion transmission and storage, while maintaining a stable capacitance.

## 4. Conclusions

In summary, ZIF-67/carbon materials were synthesized from pre-hydrolysate by one- and two-step hydrothermal processes. The results demonstrated that Co-MOF (ZIF-67) could control the morphology and porous structures of pre-hydrolysate-based carbon materials, and improved the electrochemical performance of Carbon-ZIF-67. The materials showed coral-like cross-linked structures and dandelion-like hollow structures by comprising pre-hydrolysate with ZIF-67, respectively. A hollow structure could expose more active sites and large electrolyte electrode contact areas, and short electron and charge transport paths. The cavity further prevents the accumulation of an electrochemical active center to protect them from the interference of electrolytes and the formation of solid electrolyte phase interface film layers. The superior porous structures could provide more space for charge storage and more channels for the rapid transfer of electrolyte ions. The C2-ZIF-67 showed excellent specific capacitance (400 F g^−1^, at 0.5 A g^−1^) and stability (90%, after 10,000 cycle number) due to the synergistic effect of the structures of the carbon materials and active groups of ZIF-67 with lower amounts. This work realizes the high value utilization of pre-hydrolysate and the full utilization of resources, and expands the application field of pre-hydrolysate and Co-MOFs, which provides a favorable theoretical basis and technical support for the development and utilization of bio-based carbon materials in the field of supercapacitors.

## Figures and Tables

**Table 1:** Pore structure parameters of the investigated samples.

Sample	S_BET_ (m^2^ g^−1^)	S_micro_/S_BET_ (%)	V_total_ (cm^3^ g^−1^)	Average Pore Size (nm)
C1-ZIF-67	121	11	0.209	11.9
C2-ZIF-67	235	38	0.254	8.4

**Table 2:** The specific capacitance of C-ZIF-67 and other materials.

Materials	Electrolyte	Current Density (A g^−1^)	Specific Capacitance (F g^−1^)	Ref.
Ni_3_(HITP)_2_ EDLC	TEABF_4_/ACN	0.05	111	[38]
PC-Zn	KOH	0.5	138	[39]
IM-HPC	H_2_SO_4_	0.5	236	[40]
Co_3_O_4_@Carbon Peanut shells@FeCl_3_/MgCl_2_	KOH Na_2_SO_4_	1 1	261 247	[41] [42]
Cellulose paper @Ni Lignin/single walled CNT hydrogel Co-Al-LDH@Carbon	KOH Cellulose/Li_2_SO_4_ gel H_2_SO_4_	0.2 0.5 1	268 292 300.7	[43] [44] [45]
CNTs/NCP	H_2_SO_4_	1	308	[46]
C-ZIF-67	KOH	0.5	400	This work

## Data Availability

The data presented in this study are available on request from the corresponding author.
